# Notes

**Published:** 1899-07-15

**Authors:** 


### The Institutional Workshop.

### NOTES.

An interesting little ceremony took place last week at Marlborough House, when certificates and medals were awarded, in the presence of the Prince of Wales, to 13 recipients for heroic conduct in saving life. This power of recognising gallant actions is not one of the least pleasant functions of the Chapter-General of the Grand Priory of St. John of Jerusalem.

The Parish Council of Govan have just opened a new hospital. It is connected with the old hospital by a corridor. The new hospital contains accommodation for about 112 patients in the general wards, and there is besides accommodation for maternity cases and convalescents. The whole expenditure has been about £20,000.

The munificent bequest of Mr. James Weir to the Dublin hospitals has now been distributed. The majority of the hospitals received £1,000 each at the first distribution, but the final donations by the executors have now been determined, and some of the institutions have received as much as £5,000 each, and a few a still larger sum.

As illustrating the remarks we made last week as to the cost of hospital construction, we would draw attention to the proceedings at the last meeting of the Metropolitan Asylums Board, when it was shown that what some might call the trifling alteration in a hospital plan involved in making each nursing unit consist of 15 beds instead of 12, would lead to as aving in first cost of £30,000, and an annual reduction of about £950 in the staff expenses. Mr. J. Brown moved the adoption of a report from the Works Committee upon a letter received from the Local Government Board in which they adverted to the recent conference between the Board's officers and representatives of the Managers respecting the plans of the proposed new Southern Hospital, and returned the same to the Managers in order that they might further consider the proposals made at the conference with a view to a substantial reduction in the cost of the buildings. The hospital was designed to accommodate 720 patients (with 80 additional beds for isolation cases), distributed in 30 double cottages for 24 patients each—*i.e.*, 12 in one cottage. The committee now reported that, as the result of further consideration, they had arrived at the conclusion that a material reduction, estimated by the architects at £30,000, could be made in the cost of the hospital, without detriment to its utility, if the unit of accommodation in the single cottages were increased from 12 to 15 beds. This would also permit of the staff being reduced by 12 house-mothers and six wardmaids —a reduction which represented an annual saving of about £950. They submitted recommendations to the effect that, subject to the sanction of the Local Government Board, the unit of accommodation to be provided in each single cottage at the Southern Hospital be increased from 12 to 15 beds, and that the amended plans, designed to carry out the revised scheme, be approved and forwarded to the Local Government Board for their sanction under seal. The recommendations were adopted.

Two children's wards have been added to the Sunderland Eye Infirmary by the conversion of some attic rooms on the third floor of the building. This will provide for 12 patients with the necessary offices, and adjoining nurses' quarters. The old nurses' quarters are to be occupied by adult patients.

At the meeting of the London County Council held on Tuesday the Local Government and Taxation Committee reported with reference to the question of the exemption of the London hospitals from rating, and recommended, " That, pending the report of the Royal Commission on Local Taxation, the Council do take no steps with a view of creating fresh exemptions from liability to rating." The recommendation was adopted.

An infectious diseases hospital has been opened at Kirkcaldy. It consists of two ward blocks and administrative block. There are two wards devoted to scarlet fever with 15 beds, and one ward for enteric fever, one for measles, and one observation ward. Each ward is provided with a sun-room for convalescent patients. The cost of the building and furnishing is estimated at £11,000. There are 30 beds in all.

The renovated buildings of the City Orthopædic Hospital were opened by the Duke of Cambridge last week. The alterations were very necessary, especially those appertaining to the sanitary arrangements. The cost of the improvements has been £5,000, towards which nearly £3,000 has been contributed. There is, besides, a debt of £6,000 against the hospital for the purchase of the freehold.

The alterations which are being made at the Chelsea Hospital for Women comprise the enlargement of the operating theatre so as to provide more accommodation for visitors, and the removal of the sterilising apparatus, &c., to an ante-room. The electric light also is to be installed, and arrangements are to be made for working the lift by electricity. At the same time it is proposed to repaint the interior of the hospital, and for these purposes the in-patient department will be closed till the end of September.

